# A severe acute respiratory coronavirus virus 2 (SARS-CoV-2) nosocomial cluster with inter-facility spread: Lessons learned

**DOI:** 10.1017/ice.2023.172

**Published:** 2024-05

**Authors:** Aurora E. Pop-Vicas, Laura Anderson, Gabrielle Hatas, Linda Stevens, Ashley Buys, David O’Connor, Nancy Wilson, Kasen Riemersma, Luis A Haddock Soto, Abby Richardson, Christine Clemens, Jennylynde Packham, Daniel Shirley, Nasia Safdar

**Affiliations:** 1 Division of Infectious Disease, Department of Medicine, University of Wisconsin School of Medicine and Public Health, Madison, Wisconsin; 2 Department of Infection Control and Prevention, University of Wisconsin Health University Hospital, Madison, Wisconsin; 3 Nursing Quality and Safety, University of Wisconsin Health University Hospital, Madison, Wisconsin; 4 Employee Health Services, University of Wisconsin Hospitals and Clinics, Madison, Wisconsin; 5 Department of Pathology and Laboratory Medicine, University of Wisconsin, Madison, Wisconsin; 6 University of Wisconsin Health Rehabilitation Hospital, Madison, Wisconsin; 7 William S. Middleton Memorial Veterans’ Affairs Medical Center, Madison, Wisconsin

## Abstract

**Background:**

Despite infection control guidance, sporadic nosocomial coronavirus disease 2019 (COVID-19) outbreaks occur. We describe a complex severe acute respiratory coronavirus virus 2 (SARS-CoV-2) cluster with interfacility spread during the SARS-CoV-2 δ (delta) pandemic surge in the Midwest.

**Setting:**

This study was conducted in (1) a hematology-oncology ward in a regional academic medical center and (2) a geographically distant acute rehabilitation hospital.

**Methods:**

We conducted contact tracing for each COVID-19 case to identify healthcare exposures within 14 days prior to diagnosis. Liberal testing was performed for asymptomatic carriage for patients and staff. Whole-genome sequencing was conducted for all available clinical isolates from patients and healthcare workers (HCWs) to identify transmission clusters.

**Results:**

In the immunosuppressed ward, 19 cases (4 patients, 15 HCWs) shared a genetically related SARS-CoV-2 isolate. Of these 4 patients, 3 died in the hospital or within 1 week of discharge. The suspected index case was a patient with new dyspnea, diagnosed during preprocedure screening. In the rehabilitation hospital, 20 cases (5 patients and 15 HCWs) positive for COVID-19, of whom 2 patients and 3 HCWs had an isolate genetically related to the above cluster. The suspected index case was a patient from the immune suppressed ward whose positive status was not detected at admission to the rehabilitation facility. Our response to this cluster included the following interventions in both settings: restricting visitors, restricting learners, restricting overflow admissions, enforcing strict compliance with escalated PPE, access to on-site free and frequent testing for staff, and testing all patients prior to hospital discharge and transfer to other facilities.

**Conclusions:**

Stringent infection control measures can prevent nosocomial COVID-19 transmission in healthcare facilities with high-risk patients during pandemic surges. These interventions were successful in ending these outbreaks.

Preventing nosocomial coronavirus disease 2019 (COVID-19) is a priority, and infection control guidance is continuously evolving.^1–3^ Outbreaks have occurred nonetheless,^4–7^ indicating the need to refine prevention programs. We describe a COVID-19 nosocomial cluster within an inpatient unit of a tertiary-care hospital hosting immunocompromised patients with subsequent spread to an acute rehabilitation facility during July–October 2021, when the severe acute respiratory coronavirus virus 2 (SARS-CoV-2) δ (delta) variant became dominant. This report provides further insight into preventing intra- and interfacility spread during a coronavirus disease 2019 (COVID-19) pandemic surge.

## Methods

### Healthcare settings

This study was conducted in 2 Midwest settings: (1) a 39-bed inpatient cancer ward with patients having stem-cell bone-marrow transplants, hematological and oncological malignancies, and palliative care within a 515-bed academic regional referral center and (2) an affiliated, geographically distant, 50-bed, acute-care, inpatient rehabilitation hospital. Patients in both settings were housed in individual hospital rooms. Nosocomial COVID-19 cases were defined as patients with a negative SARS-CoV-2 test on admission and a subsequent positive SARS-CoV-2 test ≥5 days into their hospital stay. The cluster investigation was considered quality improvement and was exempt from review by the institutional review board.

### Cluster investigation in the tertiary-care hospital

#### Infection control measures in place prior to cluster detection

Patients underwent SARS-CoV-2 polymerase chain reaction (PCR) testing at admission, within 24–48 hours prior to invasive procedures, and if they developed COVID-19 symptoms. Healthcare workers (HCWs) self-monitored daily and reported for free SARS-CoV-2 PCR testing if they developed any COVID-19 symptoms. Employees and HCWs with confirmed COVID-19 returned to work after 10 days if symptoms improved. Surgical-barrier masks were required for all HCWs, with disposal at the end of each shift, unless visibly soiled, damaged, or wet. Eye protection was strongly recommended but not required. Powered air-purifying respirators (PPARs) or N95 respirators, eye protection, gowns, and gloves were required during care of all patients with COVID-19 confirmed within the previous 0–20 days. Otherwise, for patients who had tested positive within the previous 21–90 days, this protective equipment was only required during aerosolized-generating procedures. Patients with confirmed COVID-19 in the previous 20 days were treated in separate COVID-19 inpatient wards with negative-pressure airflow rooms and were encouraged to wear masks when others were in their rooms. The cancer ward accommodated medical training for healthcare learners of all levels and occasionally admitted overflow immune-competent patients. Patients were allowed 1 visitor each, screened for COVID-19 symptoms and/or positive test within the prior 20 days, always wearing a mask, and not permitted to eat in the patient’s room. As of July 1, 2021, the Employee Health Services department had documentation of completed primary series for COVID-19 vaccination for 89% of all HCWs.

#### Index patient

On day 0, the infection control department was notified of a potential COVID-19 nosocomial case on the cancer ward. The patient had been in the hospital for a month, had undergone a second stem-cell transplant for underlying neutrophilic acute lymphoblastic leukemia, and was beginning to engraft. The patient, scheduled for bronchoscopy to evaluate new fevers, dyspnea, hypoxia, and radiological findings of acute lung injury with an organizing pneumonia pattern, had a positive SARS-CoV-2 PCR test during preprocedure screening. The patient’s SARS-CoV-2 isolate could not be genetically sequenced due to the amount of viral material being too low, with a subsequent negative test within a week. Although the index case status could not be confirmed by molecular typing, this patient’s case did trigger an infection control response and subsequent investigation.

#### Epidemiological investigation

Each patient with laboratory-confirmed COVID-19 had contact tracing to identify whether any SARS-CoV-2–positive HCW cared for the patient within 14 days prior to symptom onset or, for asymptomatic cases, prior to the first positive test. Strong epidemiological links were defined as evidence of an HCW being present in the patient’s room based on review of the electronic medical record, irrespective of the personal protective equipment worn. Weak epidemiological links were defined as an HCW being present on the affected inpatient unit during the outbreak period, without documented direct care for a positive case. For HCWs, high-risk exposures were defined as being present during an aerosol-generating procedure without eye protection and a fit-tested N95 respirator or PAPR in the room of a patient during the 10 days prior to patient’s diagnosis of laboratory-confirmed COVID-19.

#### Molecular typing

All available clinical specimens from positive patients and HCWs were sent for molecular sequencing, with regulatory approval from the Western Institutional Review Board (WIRB #1-1290953-1). Viral RNA isolation was performed as previously described.^8,9^ Complementary DNA generation was synthesized using a modified ARTIC Network approach.^10^ Briefly, primers for overlapping 500-bp amplicons spanning the entire genome were amplified in 2 multiplexed PCR reactions using the conditions previously described^9,10^ The 2 multiplexed PCR reactions were pooled prior to ONT library preparation. Samples were made compatible for deep sequencing using the one-pot native ligation protocol with Oxford Nanopore kit SQK-LSK109 and its Native Barcodes (EXP-NBD104 and EXP-NBD114) (Quick 2020). Samples were end repaired using the NEBNext Ultra II End Repair/dA-Tailing Module (New England Biolabs, Ipswich, MA). Samples were then barcoded using 2.5µL ONT native barcodes and the Ultra II end repair module. Samples were tagged with ONT sequencing adaptors according to the modified one-pot ligation protocol.^9^ Up to 24 samples were pooled prior to being run on the appropriate flow cell (FLO-MIN106). Sequencing data were processed using the ARTIC bioinformatics pipeline (https://github.com/artic-network/artic-ncov2019), with a few modifications. We modified the ARTIC pipeline to demultiplex raw fastq files using qcat because each fastq file is generated by the GridION (https://github.com/nanoporetech/qcat). Once a barcode reached 100,000 reads, it triggered the rest of the ARTIC bioinformatics workflow to map to the Wuhan-Hu-1 SARS-CoV-2 genome (Genbank no. MN908947.3) using minimap2. The alignment was then used to generate consensus sequences and variant calls using medaka (https://github.com/nanoporetech/medaka).

*Phylogenetic analysis.* A maximum likelihood phylogenetic tree was generated for all sequenced specimens by IQTree (http://www.iqtree.org/) using the best-fit substitution model (HKY+F+I) and 1,000 ultrafast bootstrap iterations. The length of tree branches in the horizontal axis represents genetic distance (nucleotide substitutions per site in the SARS-CoV-2 genome). Sequences in the same clade with short branch lengths are indicative of transmission clusters. Two mutations were used as the cutoff for relatedness. All putative transmission clusters identified in the phylogenetic tree were confirmed by visual inspection of the sequences. Pango lineages were assigned using the current version of Nextclade.

### Cluster investigation at the rehabilitation hospital

#### Infection control measures prior to cluster detection

Patients accepted for transfer to the rehabilitation hospital were not routinely tested for SARS-CoV-2 upon admission to the facility, although patients with a known positive SARS-CoV-2 PCR test within the previous 20 days (potentially infectious) were excluded from admission. Patients dined in their own private rooms but participated in shared rehabilitation therapies with other patients and staff. Each patient was allowed 1 visitor, and friends or relatives could accompany patients to outside medical appointments. Patients and visitors were encouraged to wear masks inside the rehabilitation facility, although compliance with this recommendation was, at times, difficult to enforce. The use of surgical masks by HCWs was required throughout the hospital, with disposal at the end of each shift or was visibly soiled, damaged, or wet. The use of face shields during patient care was required for unvaccinated HCWs only. All HCWs had access to free diagnostics via use of Abbot ID NOW COVID-19 point-of-care molecular assay, or via alternative COVID-19 testing available in their community. If positive, in addition to their rehabilitation hospital supervisor, they were required to notify University Hospital Employee Health Services, which administered a standard survey to ascertain the source of infection and to identify possible high-risk exposures in the workplace if the employee was symptomatic at work prior to testing positive. HCW vaccination was not mandatory; at the time of the cluster onset, 86% of HCWs had received at least 1 dose of a COVID-19 vaccine.

#### Cluster detection

In mid-September, the Infection Control Department at the University Hospital was notified of suspected COVID-19 nosocomial transmission at the rehabilitation hospital and was asked to assist with the epidemiological investigation. At that time, the rehabilitation hospital’s incident command had become aware of 4 patients who had tested positive for SARS-CoV-2 within 72 hours of discharge from their rehabilitation facility, and 10 HCWs had reported positive COVID-19 status within the previous week.

#### Epidemiological investigation

A thorough contact tracing investigation was attempted but was deemed insufficient given several difficulties such as accurately identifying workplace exposures retrospectively for patients no longer present in the facility, HCWs out on sick leave, and incomplete or delayed exposure data from the employee health services department. In addition, patients often shared common spaces for rehabilitation therapies and social activities, and HCWs frequently provided patient assistance that was not always documented in the electronic medical record throughout the entire facility (as opposed to specific patient room assignments within a single ward typical of acute hospital care). These realities posed significant challenges to retrospective contact tracing. Thus, the decision was made to conduct SARS-CoV-2 PCR screening of all patients residing in the facility and all HCWs whose job involved face-to-face patient interactions, to identify asymptomatic carriers and to obtain clinical specimens for molecular typing.

## Results

### Acute-care hospital cluster in the cancer ward

**Figure 1.**
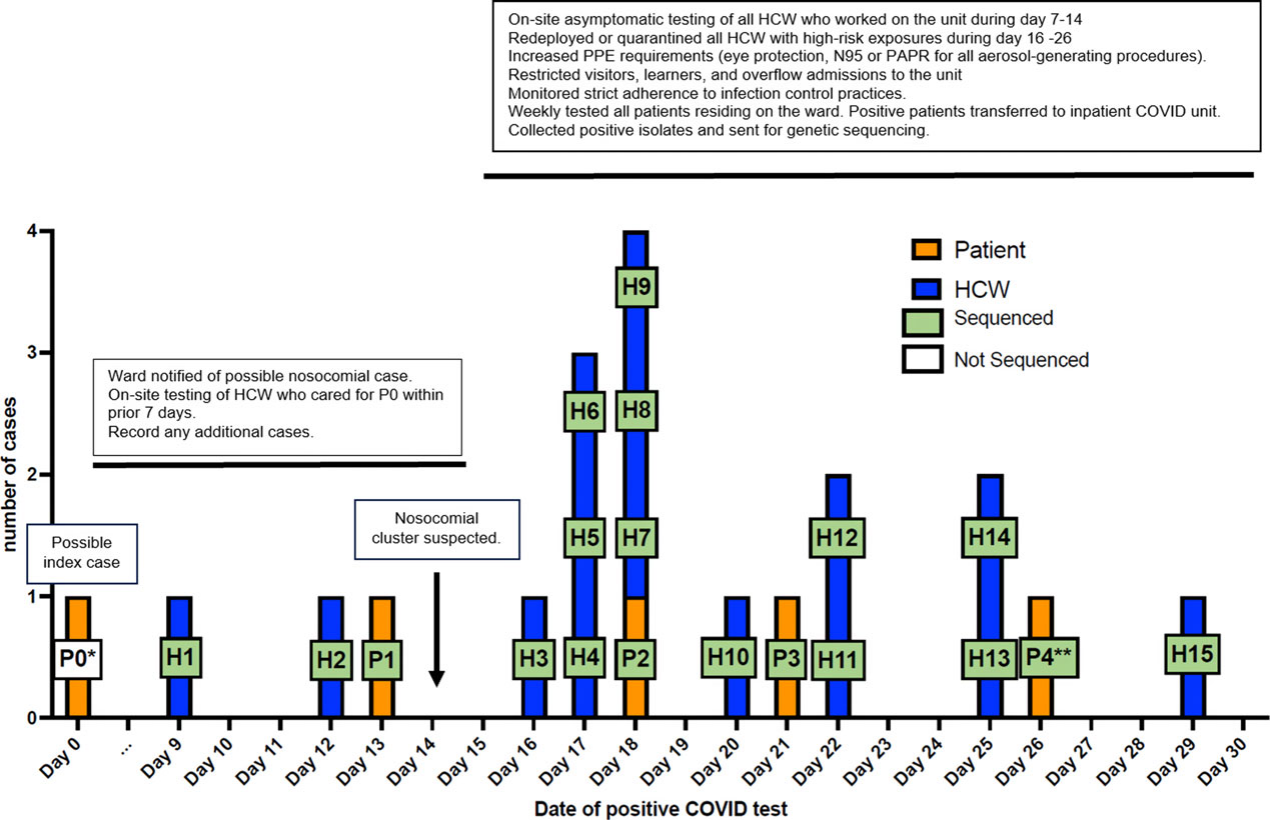
Epidemiology curve of nosocomial COVID-19 cases in the immune-suppressed ward at the University Hospital. The cluster occurred during July–August 2021. High-risk exposures were defined as being present during an aerosol-generating procedure without eye protection and a fit-tested N95 respirator or PAPR in the room of a patient diagnosed with laboratory-confirmed COVID-19 within the subsequent 10 days. *Patient 0 was diagnosed with nosocomial COVID-19 after a positive SARS-CoV-2 PCR test during prebronchoscopy screening for evaluation of dyspnea in an immunocompromised host. Although genetic sequencing was not available, it is possible that P0 was the cluster’s index case. **P4 is suspected to have been infected on the acute-care hospital immune-suppressed ward and subsequently to have become the index P1 patient in the rehabilitation hospital cluster (see Fig. 3). Because there were no known positive patients or HCWs from this unit during days 1–8, this time frame is not represented on the graph. No other nosocomial cases were identified for 8 months after day 29. Note. P, patient; H, healthcare worker.

Table 1 shows demographic and clinical details related to patients’ COVID-19 and hospital course. Of the 5 patients involved in this cluster, 2 died in the hospital and 1 died in hospice within 1 week of hospital discharge. Figure 1 shows the epidemiological curve for the 15 HCWs and 5 patients who were part of this nosocomial cluster as well as the main infection control measures implemented in response.

**Table 1.** Demographic and Clinical Details Related to COVID-19 for Cluster Patients from the Inpatient Cancer Ward and from the Rehabilitation Center

Patient	Age	Sex	Reason for Admission	Time to Positivity After Admission	COVID-19 Clinical Manifestations	COVID-19 Treatment	Comorbid Illnesses	Other Acute Issues in the Hospital	Hospitalization Outcome
**Inpatient acute-care hospital: Immune-suppressed ward**									
P0	51	F	Typhlitis and septicemia	28 days	New onset dyspnea, hypoxia, and organizing pneumonia	Remdesivir + dexamethasone + tocilizumab	Chronic neutrophilic leukemia after stem-cell transplant with graft failure	Received second stem-cell transplant	Discharged home
P1	62	M	Relapsed acute myelogenous leukemia	12 days	Fever, hypoxia, cough, diarrhea; new diffuse lung nodules; ground-glass opacities in the right lung apex, progressed to organizing pneumonia during hospitalization	Remdesivir + dexamethasone	Hypertension Chronic lower extremity venous insufficiency ulcers Obesity	Neutropenic fever, pulmonary nodules, suspected fungal infection, *C. difficile* infection	Deceased
P2	70	M	Widely metastatic disease (lung, bone with T9 pathological fracture, liver, adrenal) from non–small-cell lung cancer	17 days	Fever, tachycardia, hemoptysis; intermittent hypoxia	Remdesivir + dexamethasone	Chronic obstructive pulmonary disease	Non-ST elevation myocardial infarct; right clavicular pathological fracture	Discharged to hospice
P3	77	M	Acute decompensated heart failure, acute chronic renal failure	20 days	Progressive hypoxic respiratory failure	Dexamethasone	Multiple myeloma with pathological fractures, on chemotherapy	MRSA bacteremia with sepsis; infectious superficial thrombophlebitis; cardiorenal syndrome; paroxysmal atrial fibrillation; toxic metabolic encephalopathy	Deceased
P4^a^ (rP1 in rehabilitation)	50	F	Altered mental status (initial hospitalization)	19 days	Fever, hypotension, tachycardia, hypoxia (developed at rehabilitation hospital, 3 days prior to readmission to acute-care hospital)	Remdesivir and dexamethasone	Post-stem cell transplant for acute lymphoblastic leukemia; complicated by GVHD (skin, GI tract), PTLD (lung nodules), CMV reactivation, subdural hematomas, steroid myopathy, recurrent bacterial infections	Pancytopenia; pulmonary nodules (organizing pneumonia by biopsy, with otherwise negative workup); gliosis and white matter changes in the frontal lobe, negative infectious and hematological-oncological workup	Discharged to rehabilitation facility 12 days after initial hospitalization without COVID-19 testing upon hospital discharge and/or rehab admission; re-admitted to acute hospital 7 days later with COVID-19 infection; discharged home after second hospitalization
**Rehabilitation hospital**									
rP2	67	F	Rehabilitation after hospitalization for acute psychosis secondary to Parkinson’s disease	12 days	Asymptomatic, SARS-CoV-2–positive at discharge screening	Supportive therapy only	Parkinson’s disease, depression, hypertension, obstructive sleep apnea, asthma	None	Discharged to skilled nursing facility; remained asymptomatic
rP3	91	M	Rehabilitation after hospitalization for acute cardioembolic stroke	22 days	Readmitted to outside hospital 2 days after discharge from rehabilitation hospital; presented with falls and generalized weakness; SARS-CoV-2–positive on admission screening at outside hospital	Casirivimab and imdevimab	Prostate cancer metastatic to bone, hypertension, hyperlipidemia, peripheral vascular disease, hypothyroidism	None	Discharged to skilled nursing facility for 2 weeks, then discharged home
rP4	41	M	Rehabilitation after below knee amputation	11 days	Readmitted 3 days after discharge from rehabilitation hospital with dyspnea and diarrhea; SARS-CoV-2 positive and pneumonia; progressed to severe hypoxic respiratory failure	Dexamethasone; tocilizumab	End-stage renal disease on peritoneal dialysis, diabetes mellitus, lower extremity osteomyelitis, cirrhosis due to hepatitis B, hypertension	Non-ST elevation myocardial infarction;	Deceased
rP5	66	M	Rehabilitation after cardioembolic stroke	19 days	Mild sore throat	Supportive therapy only	Hypertension, obstructive sleep apnea, obesity, systolic heart failure	Neutropenia	Discharged home

Note. MRSA, methicillin-resistant *Staphylococcus aureus*; GVHD, graft versus host disease; PTLD, post-transplant lymphoproliferative disorder.

a P4 is suspected to have acquired infection within the 5 days before discharge to the rehabilitation hospital; she was likely the index case (rP1) of the cluster in the rehabilitation hospital.

#### Nosocomial transmission

Figure 2a shows the schematic representation of patient locations on the immune suppressed ward and their associated HCWs. Identified opportunities for transmission included a shared meal between HCWs H1 and H2 the day before HCW H1 tested positive, and several days of exposure to aerosol-generating procedures without wearing a respirator and eye protection for the multiple HCWs caring for patients 1 and 3, before these patients’ positive COVID-19 status became known. Because genetic sequencing for P0 was not available, it remains unclear whether the cluster strain was first introduced on the unit by P0 or by H1. Figure 2b shows the phylogenetic tree of the cluster cases whose clinical specimens were sequenced. As seen in the figure and the associated supplementary sequence table (ST1 online), all sequences were either identical or differed by <2 mutations, representing transmission of the same strain, identified as belonging to the SARS-CoV-2 21J (delta) clade (current Pango lineage designation AY.103).

**Figure 2a.**
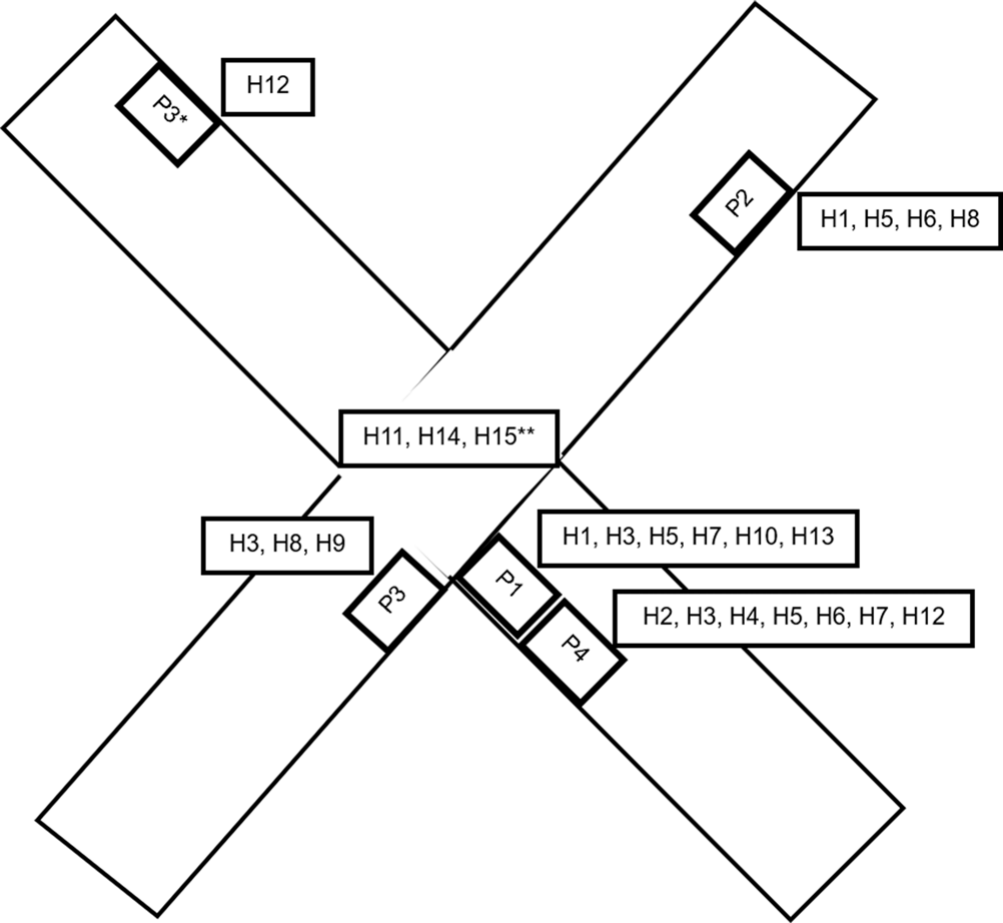
Schematic representation of the cluster on the immune suppressed ward at the university hospital. Each patient is shown with their room location and the HCWs identified to have cared for them within 10 days prior to their positive test. Patients P1 and P4 resided in adjacent rooms. *Patient P3 initially resided in a room around the corner from P1 and P4 and was moved to a room farther apart 4 days prior to positive test. **HCWs H11, H14, and H15 worked on the ward, but direct exposures to any of the other infected patients or healthcare workers were not uncovered during the epidemiological investigation. P0, the first case of nosocomial SARS-CoV-2 acquisition on the unit, is not included in this figure because genetic sequencing was not available and no HCW was SARS-CoV-2 positive in connection with this case during contact tracing. Note. P, patient; H, healthcare worker.

**Figure 2b.**
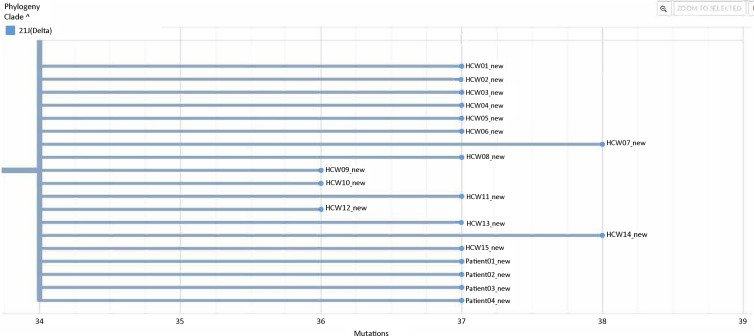
Phylogenetic analysis of the patient and HCW samples from the immune suppressed ward at the university hospital. Because there were <2 mutation differences between these samples, they were considered to represent transmission of the same strain. Although further sequencing analysis revealed single amino acid changes in HCWs H7, H11, and H14 compared to the rest of the group, this is typical of the variation within the transmission cluster of a shared strain. Note. P, patient; H, healthcare worker.

#### Response to index patient

After the index patient (P0) was identified as a nosocomial case (Fig. 1) and moved to the special COVID-19 care unit, the cancer ward was notified. The HCWs who cared for P0 within the previous 10 days were identified, and those who had not yet completed their primary vaccine series were required to test for SARS-CoV-2. This approach did not identify any SARS-CoV-2–positive HCWs in connection with P0. However, by day 14, 3 additional cases (2 HCWs and 1 patient) were identified in connection with the cancer ward, and a nosocomial cluster was strongly suspected.

#### Response to the cluster

The following measures were implemented on day 15 and remained in place for at least a month after the end of the cluster: (1) All HCWs, including learners who had been present on the cancer ward for at least 15 minutes during the prior 10 days were required to undergo SARS-CoV-2 testing made available on site at the hospital. (2) Protective eye wear (face shields or goggles) became mandatory; surgical barrier masks were to be replaced after exiting each patient room; and fit-tested N95 respirators or PAPR became required for all aerosolized generating procedures, regardless of patient’s COVID status. (3) HCWs with high-risk exposures were required to test at least twice during the course of a week. If negative and asymptomatic, they were redeployed to other hospital wards for 14 days. If positive, they were excluded from work for at least 10 days. (4) All patients on the unit underwent immediate and then serial testing weekly, regardless of symptoms, and those positive for SARS-CoV-2 were moved to a specialized COVID-19 unit. (5) Strict adherence to infection control measures, including hand hygiene and correct use of PPE at all times, was emphasized and routinely monitored. (6) Visitors were restricted from entering the unit, except for end-of-life situations. (7) Learners (ie, students from all health specialties) were no longer allowed on the unit, although medical house staff (residents and fellows) continued to work on the unit. (8) Overflow admissions of non–immune-suppressed patients were no longer accepted on the unit. The cluster ended on day 29. No further nosocomial SARS-CoV-2 acquisition was detected on that unit for at least 8 months thereafter.

### Rehabilitation center COVID-19 cluster

Of the 5 patients involved in this cluster, 1 died in the hospital, and 4 patients were discharged to a lower-level skilled nursing facility or to home (Table 1). Notably, none of the cluster patients were tested for SARS-CoV-2 while residing in the rehabilitation facility. Rather, they were diagnosed with COVID-19 due to testing positive upon admission to another facility within 48 hours of discharge from the rehabilitation hospital.

**Figure 3.**
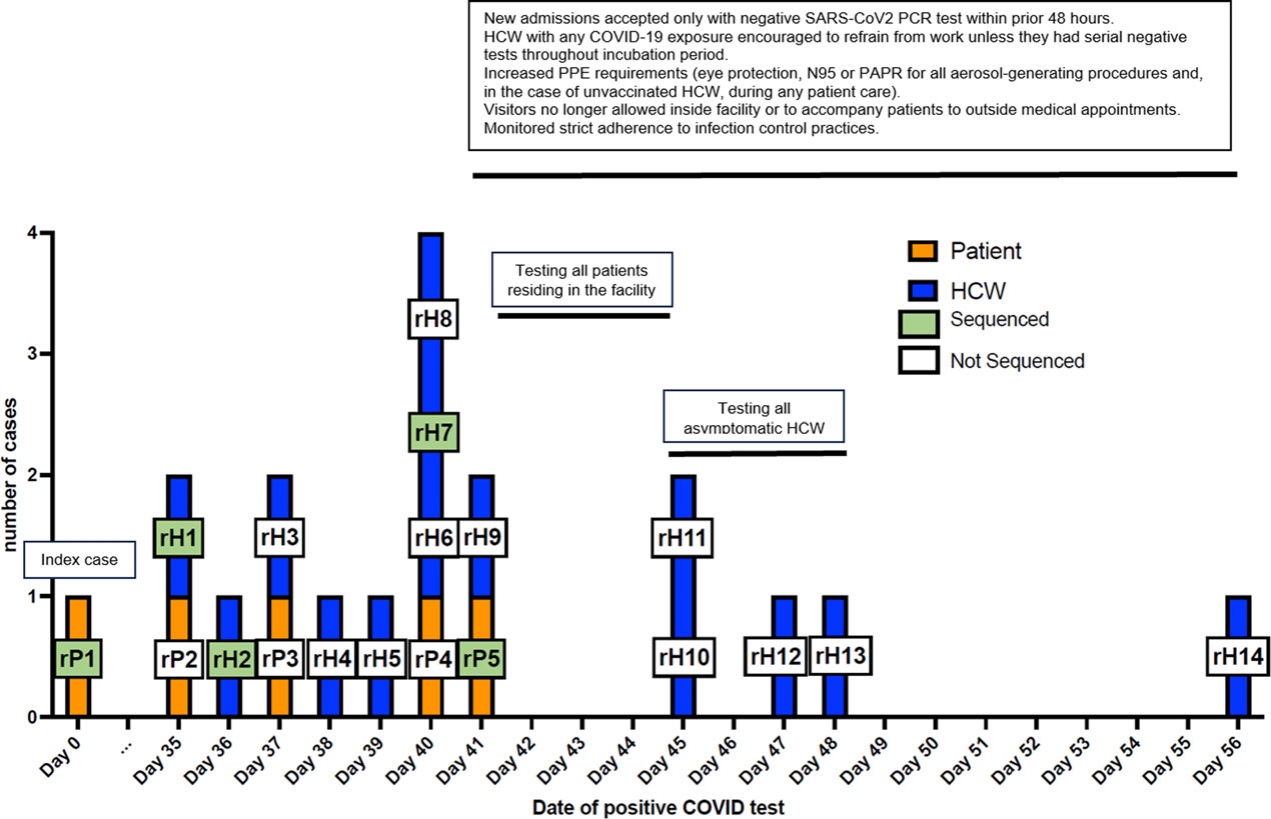
Epidemiological curve of nosocomial COVID-19 cases in the acute rehabilitation facility. The cluster was investigated during September–October 2021. After genetic sequencing results became available, patient P1 in this figure, the same as patient P4 from Figure 1, was retrospectively recognized as the common link that introduced the cluster strain into the rehabilitation facility during their admission in August. In the absence of rigorous surveillance at that time, it is likely that there was unrecognized nosocomial transmission within the facility during days 1–34 and that the size of the rehabilitation cluster has been underestimated. The isolates from the cases labeled with white squares either were not available for sequencing or could not be sequenced due to insufficient viral material, as was the case for HCWs rH10, rH11, rH12, rH13, who remained asymptomatic. HCW rH14 had community exposure, became symptomatic at home, and reported a subsequent SARS-CoV-2–positive test in the community, with clinical specimen unavailable for sequencing. Although HCW rH14 may not have been infected with the cluster strain, rH14 is included on the graph as the last positive case within a 14-day incubation period from last work shift within the rehabilitation facility. Note. rP, rehabilitation patient; rH, rehabilitation healthcare worker.

Figure 3 shows the epidemiological curve for the 15 HCW and 5 patients who tested positive for SARS-CoV-2 during timeframe of the cluster at the rehabilitation center and the implementation of the associated infection control measures. Supplementary Figure S1 (online) shows the phylogenetic tree for the few isolates from the rehabilitation facility that could be sequenced, the sequencing, and their comparison with the University Hospital isolates (Supplementary Fig. S2 online). There was a 4-nucleotide difference between the index case rP1 (same as P4 in Fig. 1) and the other isolates from the rehabilitation facility (Supplementary Sequencing Table online). However, because there was no change in the corresponding amino acids coded, these isolates from the rehabilitation facility were considered to all represent the same strain. The cluster from the rehabilitation facility was detected during September–October, ∼1 month after the end of the University Hospital cluster. Because most isolates were not available for genetic sequencing, the true extent to which the same University Hospital strain spread within the rehabilitation facility has likely been underestimated.

#### Index case and subsequent transmission

Patient 1 from Fig. 3 (ie, same as patient 4 in Fig. 1) was not initially recognized as part of the cluster in the rehabilitation facility because admission was ∼5 weeks prior to the subsequent rehabilitation cases. Once results of genetical sequencing were available, it was concluded that patient 1 could have been the index case. Because patient 1 was not tested for COVID-19 at admission to the rehabilitation center and was not isolated, ongoing but unrecognized transmission within the facility may have occurred in late August and early September.

#### Response to the cluster

Between days 45 and 48, 151 (79%) of 190 HCWs in the rehabilitation facility underwent PCR testing for COVID-19. Of these, 4 asymptomatic HCWs tested newly positive and were isolated at home on paid leave for 10 days. All patients residing in the facility during days 41–45 were also tested for COVID-19, and none were found positive. The following infection control measures were implemented concurrently: (1) Visitors were no longer allowed in the facility and could no longer accompany or transport patients to outside medical appointments. (2) Patients were accepted for admission only if they had a negative SARS-CoV-2 PCR test within 48 hours prior to arrival. (3) Vaccinated HCWs were required to wear face shields in addition to surgical face mask, and unvaccinated HCWs had to wear N95 masks with face shields. (4) N95 masks or PAPR were required for all aerosol-generating procedures. (5) Clinicians were encouraged to maintain a high index of suspicion for COVID-19 and to test any patient with symptoms compatible with infection. (6) HCWs with known exposures were strongly encouraged to refrain from working unless they tested negative for COVID-19 at least twice throughout the incubation period. (7) Strict adherence to infection control measures, including hand hygiene and correct use of PPE at all times, was emphasized and routinely monitored. After instituting these measures, no further nosocomial SARS-CoV-2 acquisition was detected for any patient in the rehabilitation facility for at least 10 months thereafter.

## Discussion

This interfacility cluster at the beginning of the SARS-CoV-2 δ (delta) variant wave of the COVID-19 pandemic in the Midwest highlights several important lessons. First, at a time when viral immunity may be increasing in the general population, initially stringent public health policies are relaxing, and the world is trying to resume normal operations, SARS-CoV-2 remains severe and often lethal in immune-suppressed hospitalized patients. As previously shown, COVID-19 mortality among cancer patients is much higher (up to 23% higher) compared to 2.3% in the general population.^11^ This risk is particularly high in hematological malignancies, with reported case fatality rates of up to 37%.^12^ Patients with lung cancer also have poor outcomes,^13^ especially with concurrent COPD.^14^ Many patients with hematological malignancies cannot mount robust immunological responses to vaccination,^15^ and vaccine effectiveness in cancer patients wanes more rapidly than in the general population.^16^ Thus, preventing nosocomial transmission to these highly vulnerable patients is vital.

Second, compared to acute healthcare settings, rehabilitation hospitals face different challenges. Patients surviving ICU hospitalizations have impaired functional mobility and cognition that require frequent, prolonged, and close interactions with HCWs to optimize recovery.^17^ HCWs frequently mix throughout the entire facility to assist with care delivery among patient rooms, ward units, and therapy spaces.^18^ Contact among patients is facilitated by group-based and shared therapy spaces.^19^ Home caregivers are often included in the rehabilitation process in preparation for discharge, and visitor compliance with recommended social distancing and/or use of personal protective equipment and/or masking may decrease, either due to pandemic fatigue or because of denial and scientific mistrust taking hold within the larger society.^20^ The infection control departments may be understaffed, and expertise may vary between for-profit and nonprofit facilities.^21^ Notably, none of the COVID-19 patients in the rehabilitation center described in this cluster were diagnosed while still residing in the facility; moreover, genetic sequencing revealed evidence of similar virus between the index patient and subsequent rehabilitation center cases >1 month apart, which implies that at least some undetected nosocomial transmission occurred within this time frame. In this context, knowing whether a patient is contagious at admission to a rehabilitation facility can be helpful in preventing nosocomial spread to vulnerable populations.^22^ Although the Society for Healthcare Epidemiology of America (SHEA) has recently recommended against routine universal SARS-CoV-2 testing of asymptomatic patients in healthcare facilities,^23^ their guidance does recognize the potentially beneficial role of admission screening in certain high-risk settings such as those providing congregate care during times of pandemic surges. This screening may be particularly important in profoundly immune-suppressed patients, who can shed viable SARS-CoV-2 virus for months after initial infection.^24^ In our experience, the implementation of multiple infection control measures, including admission screening and testing asymptomatic patients and HCWs during the outbreak, was ultimately successful in ending the cluster. Lastly, whole-genome sequencing was critical in uncovering the interfacility transmission dynamics and the epidemiological link between what initially were considered 2 separate, unrelated nosocomial clusters during a pandemic surge.

Our study had several limitations. Much of the contact tracing for the 2 clusters was retrospective in nature. We relied at least partially on review of electronic medical records and personal communication with key stakeholders from the affected units, which was likely subject to recall bias and may have failed to capture all possible exposures and cases. Because clinical specimens from many of the HCWs at the rehabilitation facility infected with SARS-CoV-2 during the cluster were not available for molecular testing, it was not possible to determine the complete extent of strain circulation within the 2 facilities. As discussed above, retrospective contact tracing in the rehabilitation facility proved challenging.

In summary, enforcing strict adherence to multiple, escalated infection control measures during pandemic surges can successfully control nosocomial SARS-CoV-2 transmission in high-risk settings.

## Supplementary material

The supplementary material for this article can be found at https://doi.org/10.1017/ice.2023.172
